# Neoadjuvant Immune-Checkpoint Blockade in Triple-Negative Breast Cancer: Current Evidence and Literature-Based Meta-Analysis of Randomized Trials

**DOI:** 10.3390/cancers12092497

**Published:** 2020-09-03

**Authors:** Daniele Marinelli, Marco Mazzotta, Laura Pizzuti, Eriseld Krasniqi, Teresa Gamucci, Clara Natoli, Antonino Grassadonia, Nicola Tinari, Silverio Tomao, Isabella Sperduti, Giuseppe Sanguineti, Andrea Botticelli, Agnese Fabbri, Claudio Botti, Gennaro Ciliberto, Maddalena Barba, Patrizia Vici

**Affiliations:** 1 Department of Clinical and Molecular Medicine, Oncology Unit, Sant’Andrea Hospital, Sapienza University, 00189 Rome, Italy; daniele.marinelli@uniroma1.it; 2 Division of Medical Oncology 2, IRCCS Regina Elena National Cancer Institute, 00144 Rome, Italy; marco.mazzotta@ifo.gov.it (M.M.); eriseld.krasniqi@ifo.gov.it (E.K.); patrizia.vici@ifo.gov.it (P.V.); 3 Medical Oncology, Sandro Pertini Hospital, 00157 Rome, Italy; teresa.gamucci@aslroma2.it; 4 Department of Medical, Oral and Biotechnological Sciences and CeSI-MeT, G. D’Annunzio University, 66100 Chieti, Italy; clara.natoli@unich.it (C.N.); antonino.grassadonia@unich.it (A.G.); nicola.tinari@unich.it (N.T.); 5 Department of Radiological, Oncological and Anatomo-Pathological Sciences, Policlinico Umberto I, ‘Sapienza’ University of Rome, 00161 Rome, Italy; silverio.tomao@uniroma1.it; 6 Biostatistics Unit, IRCCS Regina Elena National Cancer Institute, 00144 Rome, Italy; isabella.sperduti@ifo.gov.it; 7 Department of Radiation Oncology, IRCCS Regina Elena National Cancer Institute, 00144 Rome, Italy; giuseppe.sanguineti@ifo.gov.it; 8 Medical Oncology Unit B, Policlinico Umberto I, 00161 Rome, Italy; andrea.botticelli@uniroma1.it; 9 Medical Oncology Unit, Belcolle Hospital, 01100 Viterbo, Italy; agnese.fabbri@asl.vt.it; 10 Department of Surgery, IRCCS Regina Elena National Cancer Institute, 00144 Rome, Italy; claudio.botti@ifo.gov.it; 11 Scientific Direction, IRCCS Regina Elena National Cancer Institute, 00144 Rome, Italy; gennaro.ciliberto@ifo.gov.it

**Keywords:** triple-negative breast cancer, immune check point inhibitor, neoadjuvant therapy, meta-analysis

## Abstract

**Simple Summary:**

Breast cancer is a heterogeneous disease, which encompasses several subgroups of entities widely varying by clinical-pathological features. Triple negative breast cancer is characterized by a particularly aggressive biological behavior. The administration of chemotherapy has long represented the most efficacious weapon in combating triple negative breast cancer in both its initial and late phase of development. A pivot point has been recently reached throughout the approval of the immunotherapic agent atezolizumab in combination with chemotherapy as first-line treatment for programmed-death ligand 1-positive, unresectable locally advanced, or metastatic triple-negative breast cancer. Results from the registrative trial, IMpassion 130, have increasingly fueled the flourishing of studies of immune-checkpoint inhibitors in the early stage of triple negative breast cancer development. We critically interpret results from the most recent literature in light of relevant issues of methodological nature and also present a quantitative summary of data from the inherent trials. Future directions are also highlighted.

**Abstract:**

Chemotherapy based on the sequential use of anthracyclines and taxanes has long represented the most efficacious approach in the management of early-stage, triple-negative breast cancer, whose aggressive behavior is widely renowned. This standard chemotherapy backbone was subsequently enriched by the use of carboplatin, based on its association with increased pathologic complete response and efficacy in the metastatic setting. Following the results from the IMpassion130 trial, the recent approval of the immunotherapic agent atezolizumab in combination with chemotherapy as first-line treatment for programmed-death ligand 1-positive, unresectable locally advanced, or metastatic triple-negative breast cancer increasingly fueled the flourishing of trials of immune-checkpoint inhibitors in the early setting. In this work, we review the most recent inherent literature in light of key methodological issues and provide a quantitative summary of the results from phase II–III randomized trials of immunotherapic agents combined with chemotherapy in the setting of interest. Hints regarding future directions are also discussed.

## 1. Introduction

Triple-negative breast cancer (TNBC) is widely recognized as the most aggressive and lethal breast cancer (BC) subtype. It is identified by the lack of estrogen receptors (ER) and progesterone receptors (PgR), combined with the absence of both human epidermal growth factor receptor 2 (HER2) overexpression and gene amplification at the immunohistochemical (IHC) evaluation. Nonetheless, TNBC, as defined by IHC criteria, is characterized by the presence of a broad clinical and molecular heterogeneity [1,2]. Indeed, within the TNBC category, gene-expression analysis unveiled distinct molecular subtypes differing in expression signatures and ontologies, thus shedding light on the existence of the molecular mechanisms underlying complex diseases and involving different patterns. Gaining knowledge concerning such mechanisms may help to identify novel targets and efficaciously enlarge the therapeutic armamentarium currently available to TNBC patients [3,4,5].

In light of the generally high degree of responsiveness to chemotherapy, clinical management of early-stage TNBC with neoadjuvant chemotherapy is supported by multiple guidelines. Pathological complete response (pCR) after neoadjuvant chemotherapy is able to predict long-term survival outcomes and is accepted as a surrogate endpoint in early-stage TNBC clinical trials [6,7,8]. Moreover, residual cancer burden (RCB) quantification in the pathological specimen helps identify long-term outcomes, with greater residual disease associated with worse relapse-free and overall survival (OS) in the TNBC population [9].

Sequential administration of anthracycline- and taxane-based chemotherapy is the most common neoadjuvant approach, with the addition of carboplatin frequently considered in light of its association with increased pCR [10,11] and efficacy in the metastatic setting [12]. However, the most recent St. Gallen International Consensus Guidelines do not incorporate platinum-based regimens into standard-of-care neoadjuvant chemotherapy, at least in *Breast Related Cancer Antigens (BRCA*)-negative patients and in patients already receiving alkylator-, taxane-, and anthracycline-based regimens [13]. 

In recent years, the introduction of immune-checkpoint inhibitors (ICIs) in clinical practice reshaped the therapeutic landscape of several solid tumors. At its exordium, cancer may present according to different modalities in different patients, and at the single-patient level malignancies may significantly vary based on differences in the clonality of the cancer cells themselves and/or of the surrounding microenvironment [14,15]. In addition, some malignancies hold an inflammatory hallmark and occur following chronic inflammation, while others can deeply modify and/or actively involve the immune system within the net of the complex mechanisms that regulate cancer progression and metastatic spread. As a result, the interplay between the evolving actors of the human immune system and cancer may translate into widely differing outcomes, potentially spanning from complete cancer eradication to an insidious, long-term battle between these two counterparts. In the worst possible scenario, the evasion of the immune response and loss of control of cancer growth represent the ultimate outcome. Much of the current efforts in cancer immunotherapy aim to modify this latter undesirable outcome in order to achieve durable responses and survival in patients with both advanced and early-stage cancer [16,17]. 

In advanced breast cancer (mBC), single-agent administration showed modest clinical activity for programmed-death ligand 1 (PD-L1) inhibitors, with more favorable outcomes in the PD-L1 positive (+) population subset [18,19,20]. Conversely, early-phase trials of ICIs in combination with chemotherapy showed more encouraging results with significant antitumor responses, paving the way for the IMpassion130, a phase III study of the anti-PD-L1 inhibitor atezolizumab in combination with paclitaxel in the first-line setting for metastatic (m)TNBC. Patients in the combination arm showed significantly longer progression free survival (PFS) compared to those in the placebo-controlled group in both the intention-to-treat (ITT) population and the PD-L1+ subgroup (*p* = 0.002 and *p* ˂ 0.001, respectively). However, no significant OS differences were noted in the ITT interim analysis; formal testing was not performed in the PD-L1+ subset [21]. Based on the results from the IMpassion130, the Food and Drug Administration (FDA) and European Medicines Agency (EMA) granted fast approval for atezolizumab in combination with nab-paclitaxel in the first-line setting of PD-L1+ TNBC. 

In recent years, the antitumour activity of the immune checkpoint inhibitors (ICIs) in combination with chemotherapeutic agents was also intensively investigated in the neoadjuvant setting, within a frame of trials whose standard chemotherapy backbone included anthracyclines, taxanes, and/or platinum. Several authors previously reviewed the pertinent evidence [22,23,24]. For discussion and critical interpretation, we newly propose evidence from the most recent and representative studies in light of key methodological issues strictly related to each of the trials included. We also endow the reader with a quantitative synthesis of the antitumor activity estimates provided at the single-trial level through a literature-based meta-analysis.

## 2. Results

### 2.1. Results from the Literature Search

The aforementioned search yielded a total of 1431 citations. Based on the title and abstract screening and full text screening performed independently by two reviewers (D.M. and M.B.), four studies fulfilled the eligibility criteria and were thus further considered for critical discussion and quantitative data synthesis. 

### 2.2. Results from the Trials Included

The main characteristics of the studies included are shown in Table 1. 

The phase Ib KEYNOTE-173 study evaluated the addition of pembrolizumab, an anti-PD-1 monoclonal antibody (mAb), to a backbone of taxane- and anthracycline-based chemotherapy [25]. This study enrolled 60 patients and allocated them to six cohorts of ten patients each. All patients received a run-in dose of pembrolizumab prior to chemotherapy initiation, and patients in five out of the six cohorts received carboplatin in addition to a nab-paclitaxel and pembrolizumab backbone; all cohorts were subsequently treated with doxorubicin/cyclophosphamide in combination with pembrolizumab. In more detail, weekly nab-paclitaxel 125 mg/m^2^ was administered to patients in cohort A. In cohorts B through D, weekly nab-paclitaxel 100 mg/m^2^ (cohort B) or 125 mg/m^2^ (cohorts C and D) were administered in association with carboplatin, with the area under the curve (AUC) being 6, 5, and 2, respectively. Patients in cohorts E and F received weekly paclitaxel 80 mg/m^2^ and carboplatin. The AUC of choice were 5 and 2 for cohorts E and F, respectively. Pembrolizumab was administered for nine cycles before surgery in all study cohorts. Only two cohorts graduated for the recommended phase II dose threshold of dose-limiting toxicities, i.e., Cohort A (weekly nab-paclitaxel 125 mg/m^2^) and Cohort E (weekly paclitaxel 80 mg/m^2^ plus carboplatin AUC5 every three weeks), respectively. Overall, most treatment-related adverse events (AEs) were consistent with previously reported data for single-agent or combination therapy. Pembrolizumab was discontinued because of treatment-related AEs in 18% of the study population, with most presumed immune-related AEs being mild. One patient from cohort D (weekly nab-paclitaxel 125mg/m^2^ plus weekly carboplatin AUC2) died because of neutropenic septic shock. Pathologic complete response (defined as ypT0/ypN0) in the whole population was 60%, with no significant differences among the study cohorts; event-free survival (EFS) at 12 months was numerically higher in patients with pCR and in those who received platinum. Pretreatment PD-L1 IHC scores were reported as a combined positive score (CPS) and assessed in tumor cells, macrophages, and lymphocytes; CPS PD-L1 was ≥1% in 78% of patients and PD-L1 positivity was associated with increased rates of pCR, as well as higher pretreatment and on-treatment stromal tumor-infiltrating lymphocytes (sTILs). A moderate-to-strong correlation was also noted between PD-L1 and sTILs; moreover, a single run-in dose of pembrolizumab led to a mean absolute increase of sTILs of 11%.

The I-SPY2 phase II trial provided an adaptive randomization engine to assign participants to one of the multiple ongoing neoadjuvant cohorts, comparing each experimental cohort with a shared control arm; its platform allowed new agents to “graduate” within specific molecular signatures on the basis of a predetermined, pCR-based level of efficacy, with pCR being the primary outcome measure in the trial [26,27]. Following randomization of 69 patients to the pembrolizumab experimental arm, pembrolizumab graduated in both the HER2-signature and TNBC signature, thus reaching the predefined threshold of 85% likelihood of success in a subtype-specific, hypothetical, 300-patient, 1-to-1, confirmatory phase 3 trial. The pCR rates adjusted for subtype and time were 44% and 60% in HER2- and TNBC patients, respectively, with higher raw pCR rates (46% and 68% in HER2- and TNBC patients, respectively). However, among the 69 patients in the pembrolizumab cohort, only 29 participants were diagnosed with TNBC. Moreover, the control arm received weekly paclitaxel followed by doxorubicin/cyclophosphamide, referring to a common standard-of-care shared with HR+/HER2- disease that may be suboptimal in the TNBC subtype. Actually, the raw pCR rate reported for participants in the control arm was 23% in survival analysis (EFS). No significant differences emerged in reference to the HER2- and TNBC signature, with all patients achieving pCR demonstrating excellent outcomes. 

The phase III KEYNOTE-522 randomized, double-blind trial allocated 1174 stage II–III TNBC patients according to a 2:1 ratio to neoadjuvant/adjuvant treatment with pembrolizumab or placebo in combination with a chemotherapy backbone of weekly paclitaxel (80 mg/m^2^) plus carboplatin for four cycles, followed by 90 mg/m^2^ epirubicin plus 600 mg/m^2^ cyclophosphamide every three weeks [28]. Postoperative treatment consisted of nine cycles of pembrolizumab/placebo. The trial coprimary endpoints were pCR (defined as ypT0/Tis ypN0) and EFS in the ITT population. At the second interim analysis, the experimental arm showed significantly higher pCR rates (64.8% vs. 51.2%, *p* < 0.001), reaching the prespecified alpha of *p* = 0.003. In pCR subgroup analysis, pembrolizumab maintained its benefit versus placebo independently of PD-L1 status. Notably, pCR rates were considerably lower in PD-L1- patients than in their PD-L1+ counterparts (45.3% and 30.3% vs. 68.9% and 54.9% in PD-L1- and PD-L1+ patients, respectively), suggesting a prognostic role for PD-L1 CPS. Survival analysis included only 104 of the 327 events expected at the final analysis, with 91.3% of patients in the pembrolizumab arm and 85.3% in the control arm being event-free at 18 months (stratified HR = 0.63, 95% CI, 0.43 to 0.93). Overall, the KEYNOTE-522 trial confirmed statistically significant and clinically relevant benefits with the addition of pembrolizumab to a chemotherapy backbone in the neoadjuvant treatment of early-stage TNBC. However, the trial protocol did not permit the administration of adjuvant capecitabine, which demonstrated significant disease-free survival (DFS) and OS benefit in TNBC patients who did not achieve pCR after neoadjuvant chemotherapy in the CREATE-X trial [29]. Results from the latter were recently strengthened by data presented at the 2020 American Society of Clinical Oncology (ASCO) Annual Meeting concerning the use of maintenance therapy with metronomic capecitabine for one year in operable TNBC following standard treatment. Hazard ratios for DFS and distant disease-free survival (DDFS) were 0.63 (*p* = 0.027). and 0.56 (*p* = 0.016), respectively. However, no evidence of significantly improved five-year OS was observed for patients allocated to the intervention arm (HR, 0.74, *p* = 0.203) [30]. Even though results from clinical trials regarding the implementation of capecitabine in early TNBC were not always consistent [31,32,33], routine clinical care of TNBC not achieving pCR after neoadjuvant chemotherapy often embraces adjuvant treatment with capecitabine. Moreover, a recent meta-analysis presented at the 2019 San Antonio Breast Cancer Symposium highlighted more pronounced, significant benefits in terms of DFS and OS from the addition of capecitabine to neoadjuvant chemotherapy in the TNBC subtype [34]. 

The phase II, proof-of-concept trial GeparNuevo randomized 174 patients with early-stage TNBC to preoperative durvalumab (anti-PD-L1 mAb) or placebo in combination with neoadjuvant chemotherapy with weekly nab-paclitaxel followed by epirubicin/cyclophosphamide [35]. The primary endpoint of the study was pCR rate after neoadjuvant therapy. Overall, 117 out of 174 patients were treated according to a lead-in window phase with one durvalumab injection two weeks before the start of chemotherapy. In the overall population, no significant differences were noted in the primary outcome measure, with 53.4% and 44.2% of patients randomized to durvalumab or placebo, respectively, achieving pCR (sTILs-adjusted Odds Ratios (OR) = 1.53, *p* = 0.182). No interactions were found between sTILs and treatment. However, higher sTILs predicted pCR in the overall cohort and demonstrated similar effects both in the durvalumab and placebo arms, while increased postwindow immune-TILs trended toward pCR prediction in the durvalumab arm (*p* = 0.085). Moreover, durvalumab achieved more pCR than placebo in patients in the window cohort (interaction *p* = 0.048). Of 158 PD-L1-evaluable patients, 138 were PD-L1+. In this group, pCR rates were numerically higher. 

### 2.3. Meta-Analysis of Randomized Trials 

Results from the three randomized clinical trials, i.e., KEYNOTE-522, I-SPY2, and GeparNuevo, were included in the meta-analysis in reference to the pCR endpoint. We exclusively considered data regarding patients with TNBC in this meta-analysis. 

A total of 883 patients were included, of whom 366 (41.5%) received standard neoadjuvant chemotherapy and 517 (58.5%) received neoadjuvant chemotherapy combined with ICIs. Overall, 429 (82.9%) of 517 patients received pembrolizumab, while 88 (17.1%) received durvalumab as an immunotherapic agent. Off the 366 patients in the control arm the 517 patients in the experimental arm, 201 (54.9%) and 401 (77.5%) received carboplatin, respectively. Data on germline *BRCA* status were not available for participants from these three studies. 

**Figure 1:**
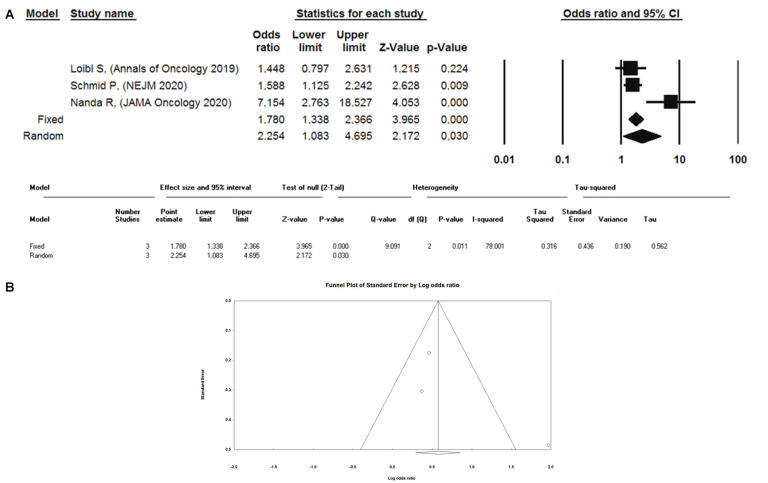
Meta-analysis of randomized trials (**A**). Funnel plot (**B**).

Primary data regarding pCR were available for 485 (54.9%) of 883 patients, i.e., 326 (63.1%) of 517 in the experimental group and 159 (43.4%) of 366 in the control group. The inherent OR and 95% Confidence Interval (CI) from the random effects model were 1.78 and 1.34–2.3, respectively. Heterogeneity was significant. Results from the fixed effect model are also shown (Figure 1A). Based on the visual inspection of the funnel plot, publication bias cannot be excluded (Figure 1B). 

### 2.4. Immune-Related Features in TNBC

As previously mentioned, significant clinical and molecular heterogeneity is known to characterize TNBC. Gene-expression-based analysis defined multiple classifications of heterogeneous molecular subtypes with significant prognostic and predictive implications in TNBC [3,36,37,38,39]. 

Bareche et al. recently addressed the topic of the remarkable heterogeneity of the tumor microenvironment (TME) among different RNA-based TNBC subtypes [40]. These authors described how immunomodulatory (IM) TNBC was predominantly associated with tumor immune-response signatures, while luminal-androgen receptor (LAR) and mesenchymal (M) subtypes were associated with low immune scores. Moreover, looking into CD8+ TIL spatial distribution, IM tumors displayed the highest prevalence of the fully inflamed (FI) pattern, with basal-like (BL) tumors predominantly associated with a stroma-restricted CD8+ TIL distribution pattern. In addition, FI tumors were enriched for medullary histology and small tumors, while margin-restricted (MR) cancers were enriched for low-grade tumors; the prognostic relevance of spatial distribution was also confirmed, with FI tumors exhibiting higher ten-year OS rates. The same research group previously described how different genomic landscapes were associated with specific TNBC subtypes [36], thus defining clinically relevant implications in TNBC subtype definition. Triple-negative breast cancer subtypes demonstrated an overall significant heterogeneity in immune cell composition, with the IM subtype enriched with adaptive immune cells and other subtypes showing predominant innate immune-response markers [41]. Among the key immune targets, IM tumors were enriched in most targets and characterized by balanced immune-stimulatory and immune-negative targets; other subtypes such as M were negatively associated with the largest proportion of immune targets. Again, the tumor border demonstrated a pivotal role in immune inhibition, characterized by specific gene expression profiles both in immune and tumor cells [42]. Moreover, expression of immunomodulatory proteins such as PD-1, PD-L1, and indoleamine 2,3-dioxygenase (IDO) by tumor and/or immune cells correlated with the overall spatial architecture of the tissue, leading to distinction between mixed and compartmentalized tumors, with both showing peculiar gene expression, spatial distribution, and prognosis.

Among the most common single-gene mutations, BRCA somatic/germline gene mutations display high frequencies in TNBC. While causing high sensitivity to DNA-damaging agents such as platinum-based chemotherapy and poly-(ADP-ribose)-polymerase (PARP) inhibitors, BRCA-associated tumors were found to display higher mutational load, TILs, and expression of immunomodulatory genes [43], and tumors with defects in the DNA-repair pathway displayed higher CD4/CD8 T-cell infiltrates [44]. However, different degrees of immunogenicity were described in BRCA-associated TNBC. Independent prognostic significance for tumor-associated inflammation was outlined in BRCA-associated TNBC [45], along with the interplay between BRCA1/2 defects and homologous recombination deficiency (HRD) scores, even in the presence of higher predicted neoantigen load, which was shown to dictate overall tumor immunogenicity [46]. 

The raw bulk of lymphocyte infiltration significantly impacts both advanced and early-stage TNBC. Stromal TILs demonstrated prognostic value in early-stage TNBC patients who did not receive adjuvant chemotherapy, with higher scores associated with improved DFS and OS in multivariate analysis [47]. A patient-level meta-analysis of randomized clinical trials and large retrospective hospital series included data from 2148 patients who received anthracycline- or anthracycline/taxane-based adjuvant chemotherapy [48]. Higher sTILs were associated with older age and higher histological grade, and 10% relative increment in sTILs was associated with improved invasive DFS, DDFS, and OS. The authors also reported a lack of significant interaction between sTILs score and treatment (chemotherapy; anthracyclines vs. anthracyclines/taxanes). Similar evidence was described when evaluating intratumoral TILs (iTILs). A multivariate model including standard clinicopathologic variables confirmed the prognostic role of sTILs in chemotherapy-treated, early-stage TNBC patients. Among patients with residual invasive disease (RD) after neoadjuvant chemotherapy for TNBC, increasing levels of sTILs were associated with improved relapse-free survival (RFS) and OS [49] This analysis was confined to patients with poor prognostic features due to the strict inclusion of patients not achieving pCR. While the overall prognostic role of sTILs was confirmed, the authors outlined significant interactions with residual cancer burden (RCB) classes, as TILs demonstrated a significant prognostic effect in residual cancer burden II and no prognostic effect in RCB I/III. As the authors stated, it can be speculated whether increased residual tumoral tissue in the surgical specimen is associated with specific characteristics of immune infiltrates or enrichment of specific downregulators of the immune response, such as FOXP3+ T-cells. Pretreatment sTIL effects on pCR, DFS, and OS were also evaluated in a combined analysis of multiple neoadjuvant clinical trials from the German Breast Group [50]. High sTILs were more frequent in TNBC than in other molecular subtypes and were predictive of increased likelihood of pCR both overall and in the TNBC subtype. While prognostic for DFS and OS in univariate analysis in the TNBC, high sTILs were not associated with survival outcome in a multivariate model of pCR. 

Specific immune markers, such as CD73 expression, showed significant prognostic impact in early-stage TNBC [51], therefore, synergistic combinations with ICIs are being tested in ongoing trials. Moreover, tissue-resident memory (TRM) T-cells were also associated to a relevant extent with a prognostic role in early-stage TNBC, and an RNA-based TRM T-cell signature was able to predict pembrolizumab efficacy in the KEYNOTE-086 trial [52,53].

## 3. Discussion

Immune-checkpoint inhibition is likely to play a pivotal role in changing the treatment landscape of early TNBC in the short term. Consistent results in support of the efficacy of anti-PD-(L)1 agents combined with chemotherapy recently emerged from three phase I/II trials, further confirmed by a randomized, double-blind phase III trial, which showed a significant and clinically relevant benefit from pembrolizumab in the neoadjuvant setting, although a longer follow-up is needed to better outline the actual survival benefit.

Our meta-analysis provides a quantitative synthesis of data from three randomized trials of ICIs combined with chemotherapy in early TNBC, whose results were published at the time of writing. Based on the proposed metrics, heterogeneity was shown to be relevant. At the same time, the low number of studies included represents an objective limitation to reliable subgroup and sensitivity analyses. However, some considerations must be undertaken when considering punctual evidence from any of the three trials which contributed data to the meta-analysis. Results from the KEYNOTE-522 and GeparNuevo led to similar OR point estimates, while findings from I-SPY2 significantly diverged, leaving ample room for the hypothesis of a driving role of the results from the latter study in impacting the summary effect measure and heterogeneity. The interpretation of I-SPY2 results in a broader TNBC context underlies the inherent trial limitations, i.e., (i) due to the prespecified statistical plan, the actual TNBC population is much smaller than in other trials included in our analysis, and (ii) the primary endpoint (pCR) analysis in the TNBC population could be significantly flawed by an underperforming control arm (raw pCR rate of 23%), even when compared with historical, nonplatinum-containing regimens (11). A further limitation to our work is represented by the evidence of asymmetry at the visual inspection of the funnel plot, where publication bias cannot be excluded. This may limit the reliability of our results as it may yield overestimated effects. However, in a recent work from van Aert, publication bias was estimated as ranging from no bias to only 5%, with statistically nonsignificant effect sizes being published in both psychology and medicine [54].

It is appropriate to outline major points still under debate. Triple-negative breast cancer is often associated with aggressive clinical behavior and early relapse, often affecting young women with a harsh impact on personal and social life. However, a relatively small fraction of immunohistochemistry (IHC)-classified TNBC is significantly less aggressive, with excellent clinical outcomes even in the absence of chemotherapy [41]. Moreover, androgen-receptor (AR) positivity characterizes 10–15% of TNBC [3,4], and is often associated with clinicopathological features resembling HR+/HER2- breast cancer (BC), with frequent association with older age and late relapse [55,56]. Particularly in elderly patients, a neoadjuvant taxane and platinum-based, anthracycline-free chemotherapy regimen showed efficacy and manageable toxicity in TNBC. In a trial from Kern and coauthors, six cycles of docetaxel (75 mg/m^2^) and carboplatin AUC 6 every three weeks were administered to patients with a reduced cardiac output or those aged 65 years and older. The authors reported fairly high rates of pCR (50%), two- and five-year DFS (96.7 and 85.7%), and two- and five-year OS (96.7 and 89.7%). Grade 3/4 toxicities were rare. No relevant association was observed between age at diagnosis and toxicity frequency or severity [57]. It is therefore of primary importance to determine if the assumption made regarding the previously discussed results is applicable to the overall body of HR-/HER2- diseases or, more plausibly, should be incorporated in a more granular TNBC landscape. At the second interim analysis, investigators from the KEYNOTE-522 study described a significant, consistent benefit from pembrolizumab over placebo both in the PD-L1+ and the PD-L1- subgroups, with about 80% of the study population classified as PD-L1+; this percentage was consistent with other reports. Lower rates of pCR were confirmed in the PD-L1- population, with no significant treatment interactions. Within the IMpassion130 study population of advanced TNBC patients, only around 40% were classified as PD-L1+ (20). While the PD-L1 scoring system used in the two studies was different and showed inconsistency when directly compared [58], it is plausible that advanced TNBC may be enriched in more aggressive features, such as PD-L1 negativity. It is crucial to better understand the role of PD-L1 both as a predictor of outcome in the early stage and as an inclusion criterion in the advanced setting. Moreover, none of the patients treated in the IMpassion130 study received previous treatment with an anti-PD-(L)1; it is therefore unknown whether retreatment with immunotherapy yields the same benefit in TNBC patients who relapse after neoadjuvant pembrolizumab. KEYNOTE-522 also used a chemotherapy backbone consisting of anthracyclines/cyclophosphamide and paclitaxel/carboplatin. While the addition of carboplatin demonstrated significant higher rates of pCR in TNBC [11], regardless of the administration schedule, neoadjuvant carboplatin in combination with taxanes was associated with non-negligible bone marrow toxicity and significant discontinuation rates [59,60]. This invites a proper clinical evaluation when discussing multidrug regimens, particularly in fragile, older patients. 

From an economic standpoint, novel drugs in recent years led to significant therapeutic improvements in most solid tumors. However, financial toxicity is also associated with large-scale implementation of biological agents [61]. While regulatory agencies from some countries granted approval to expensive drugs in case of significant therapeutic efficacy, other agencies, such as the National Institute for Health and Care Excellence (NICE), recommended against atezolizumab/nab-paclitaxel as a first-line treatment of advanced TNBC because of cost-ineffectiveness, even though it is considered a life-extending treatment [62]. The economic implications of large-scale treatment with pembrolizumab in early-stage, TNBC demand further consideration. 

## 4. Materials and Methods 

A literature review was conducted in PubMed (last update in July 2020) [63]. Both text words and the Medical Subject Headings (MeSH) thesaurus were adopted to perform a tailored search strategy, which combined terms related to “immune check point inhibitor”, “neoadjuvant therapy”, and “triple negative breast cancer”. The reference lists of the articles retrieved and proceedings from relevant international cancer meetings in the last five years were also screened to identify additional citations. Phase I–III trials of TNBC patients treated with neoadjuvant ICIs in combination with chemotherapy were considered for inclusion. Trials of breast cancer subtypes other than TNBC were considered eligible if the inherent results were separately reported by molecularly defined subgroups. 

Pathologic complete response was chosen as a potential endpoint of reference for the meta-analysis, which included results from randomized trials only. Primary data from odds ratios (OR) and 95% confidence intervals (CIs) of pCR following neoadjuvant therapy were quantitatively synthesized using the Comprehensive Meta-Analysis Software, version v.2.0 (CMA, Biostat, Englewood, NJ, USA). Statistical significance was set at *p* ˂ 0.05. The I2 and P statistics were used to test statistical heterogeneity among the studies included. An I2 value above 50% was considered representative of considerable heterogeneity across the included studies [64]. Independently of the degree of heterogeneity observed, a random-effect model was identified as the most appropriate approach. Results were also derived from a fixed-effect model. A funnel plot was used for publication bias assessment.

## 5. Conclusions

The available evidence points toward clinical implementation of immunotherapy in early-stage TNBC; nonetheless, its precise prognostic impact needs to be further analyzed with more mature survival analysis and with further data for the outcome of pCR. Moreover, the biological landscape of TNBC remains multifaceted, and its impact on the clinical management of HR-/HER2- diseases needs to be further refined.

## Figures and Tables

**Table 1:** Main characteristics and pathologic complete response (pCR) rates of clinical trials with ICIs in early-stage TNBC.

Study	Treatment	pCR Rates (N. of pts)
KEYNOTE-173, open label, phase Ib	Cohort A: P/nab-pac→P/AC Cohort B: P/nab-pac/Cb q3wks→P/AC Cohort C: P/nab-pac/Cb q3wks→P/AC Cohort D: P/nab-pac/Cb q1wk→P/AC Cohort E: P/pac/Cb q3wks→P/AC Cohort F: P/pac/Cb q1wk→P/AC	60% (6/10) 60% (6/10) 80% (8/10) 60% (6/10) 30% (3/10) 50% (5/10)
I-SPY2, randomized, phase II	Exp. arm: P/pac→P/AC Control arm: pac→AC	68% (19/28) 23% (18/79)
KEYNOTE-522, randomized, phase III	Exp. arm: P/Cb/pac→P/AC or P/EC, adj. P Control arm: plac/Cb/pac→plac/AC or plac/EC, adj. plac	64.8% (260/401) 51.2% (103/201)
GeparNuevo, randomized, phase II	Exp. arm: D→D/nab-pac→D/EC Control arm: plac→plac/nab-pac→plac/EC	53.4% (47/88) 44.2% (38/86)

ICIs: immune-checkpoint inhibitors; TNBC: triple-negative breast cancer; P: pembrolizumab; nab-pac: nab-paclitaxel; AC: doxorubicin/cyclophosphamide; Cb: carboplatin; pac: paclitaxel; D: durvalumab; adj: adjuvant; plac: placebo; wk/wks: week/s; q: every.
